# Impact of glucose metabolism abnormalities on live birth rate in South-East Asian women with polycystic ovary syndrome

**DOI:** 10.1093/hropen/hoag012

**Published:** 2026-02-17

**Authors:** Nam T Nguyen, Vu N A Ho, Toan D Pham, Minh H N Nguyen, Duy L Nguyen, Ho L Le, Khanh T Q Le, Luong D Ly, Mai T N Tran, Tuong M Ho, Robert J Norman, Lan N Vuong

**Affiliations:** IVFMD, My Duc Hospital, Ho Chi Minh City, Vietnam; HOPE Research Center, My Duc Hospital, Ho Chi Minh City, Vietnam; IVFMD, My Duc Hospital, Ho Chi Minh City, Vietnam; HOPE Research Center, My Duc Hospital, Ho Chi Minh City, Vietnam; HOPE Research Center, My Duc Hospital, Ho Chi Minh City, Vietnam; HOPE Research Center, My Duc Hospital, Ho Chi Minh City, Vietnam; HOPE Research Center, My Duc Hospital, Ho Chi Minh City, Vietnam; IVFMD, My Duc Hospital, Ho Chi Minh City, Vietnam; HOPE Research Center, My Duc Hospital, Ho Chi Minh City, Vietnam; IVFMD, My Duc Hospital, Ho Chi Minh City, Vietnam; HOPE Research Center, My Duc Hospital, Ho Chi Minh City, Vietnam; HOPE Research Center, My Duc Hospital, Ho Chi Minh City, Vietnam; Department of Physiology, University of Health Sciences, Vietnam National University Ho Chi Minh City, Ho Chi Minh City, Vietnam; Endocrine Clinic, My Duc Hospital, Ho Chi Minh City, Vietnam; HOPE Research Center, My Duc Hospital, Ho Chi Minh City, Vietnam; Endocrine Clinic, My Duc Hospital, Ho Chi Minh City, Vietnam; IVFMD, My Duc Hospital, Ho Chi Minh City, Vietnam; HOPE Research Center, My Duc Hospital, Ho Chi Minh City, Vietnam; Robinson Research Institute and Adelaide Medical School, University of Adelaide, Adelaide, SA, Australia; Department of Obstetrics and Gynaecology, University of Medicine and Pharmacy at Ho Chi Minh City, Ho Chi Minh City, Vietnam

**Keywords:** polycystic ovary syndrome, glucose metabolism, live birth, infertility, oral glucose tolerance test, South-East Asian women

## Abstract

**STUDY QUESTION:**

Is there a difference in live birth rates at 24 months between infertile women with polycystic ovary syndrome (PCOS) who have normal versus abnormal glucose metabolism?

**SUMMARY ANSWER:**

Abnormal glucose metabolism did not significantly reduce live birth rates but was associated with increased obstetric complications.

**WHAT IS KNOWN ALREADY:**

Women with PCOS are often at increased risk of glucose metabolism disorders. However, evidence about the impact of these disorders on pregnancy outcomes remains limited, particularly in Asian populations.

**STUDY DESIGN, SIZE, DURATION:**

This prospective cohort study was conducted at a reproductive care centre in Vietnam from June 2020 to August 2024. A total of 1208 women were enrolled.

**PARTICIPANTS/MATERIALS, SETTING, METHODS:**

Eligible participants were infertile women aged 18–40 years diagnosed with PCOS (Rotterdam criteria). Comprehensive assessments included medical history, anthropometric measurements, endocrine evaluations, fasting plasma glucose (FPG), glycosylated haemoglobin (HbAlc), and oral glucose tolerance tests (OGTT). Participants were categorized into normal or abnormal glucose metabolism groups and monitored for live birth outcomes at 24 months from the first visit.

**MAIN RESULTS AND THE ROLE OF CHANCE:**

Live birth rates at 24 months were comparable between women with normal versus abnormal glucose metabolism (52.7% vs 48.2%, *P* = 0.12). However, obstetric complications, including gestational diabetes mellitus (15.2% vs 28.0%, *P* < 0.001) and hypertensive disorders of pregnancy (2.3% vs 9.0%, *P* < 0.001), were more common in the group with abnormal glucose metabolism. In women who conceived naturally, greater waist circumference and higher Homeostatic Model Assessment of Insulin Resistance index were significantly associated with lower odds of live birth, whereas the presence of hyperandrogenism was associated with higher odds of live birth. No factors were significantly associated with live birth in the group that conceived via ovulation induction plus IUI. In the group that conceived through IVF or IVM, a higher BMI was significantly associated with a lower live birth rate.

**LIMITATIONS, REASONS FOR CAUTION:**

This single-centre study was conducted exclusively on infertile women from South-East Asia who had PCOS, potentially limiting generalizability to other populations. Additionally, metabolic assessments were only performed at baseline, preventing evaluation of longitudinal changes and their dynamic effects on reproductive outcomes.

**WIDER IMPLICATIONS OF THE FINDINGS:**

While no significant difference in live birth rates was observed between PCOS women with normal and abnormal glucose metabolism, the abnormal glucose metabolism group experienced higher rates of gestational complications. These findings underscore the importance of preconception metabolic screening and tailored fertility strategies that include targeted interventions to optimize reproductive and maternal outcomes in women with PCOS.

**STUDY FUNDING/COMPETING INTEREST(S):**

This study was supported by My Duc Hospital. Lan N. Vuong reports funding from the Vietnam National Foundation for Science and Technology Development (NAFOSTED; grant number FWO.108-2022.01); Merck: Speaker and conference fees; Merck Sharp and Dohme: speaker and conference fees as well as a grant; Ferring: speaker, conference, and scientific board fees outside the submitted work. All other authors declare no conflicts of interest.

**TRIAL REGISTRATION NUMBER:**

NCT04364087.

WHAT DOES THIS MEAN FOR PATIENTS? Women with polycystic ovary syndrome (PCOS) often have problems with how their body controls sugar, such as prediabetes or type 2 diabetes. These problems may not only affect the chance of having a baby but also raise the risk of complications in pregnancy.

In this study, more than 1200 Vietnamese women with PCOS were followed for 2 years. Some became pregnant naturally, while others used fertility treatments such as ovulation induction with intrauterine insemination (IUI), or more advanced treatments like *in vitro* fertilization (IVF) and *in vitro* maturation (IVM). We found that women with abnormal blood sugar control had similar chances of having a baby compared to women with normal blood sugar levels, no matter which method was used to conceive. However, those with abnormal blood sugar control had more pregnancy complications, including gestational diabetes and high blood pressure.

This means that all women with PCOS should have their blood sugar checked before trying for a baby. Early detection and care may not change the chance of getting pregnant, but it can reduce risks during pregnancy and improve health for both mother and child.

## Introduction

Polycystic ovary syndrome (PCOS) is a systemic neuroendocrine–metabolic disorder that affects 10–13% of reproductive-aged women worldwide (Teede *et al.*, 2023). Heterogeneity is one of the hallmarks of PCOS, with different clinical phenotypes, symptoms, severity, and consequences, all of which are influenced by environmental, genetic, and epigenetic factors (Barlampa *et al.*, 2021). Although not included in any diagnostic criteria, intrinsic insulin resistance (IR) is a key pathophysiological element of PCOS, occurring in 44–85% of cases, including those who have a normal body weight (Diamanti-Kandarakis and Dunaif, 2012; Stepto *et al.*, 2013). Differences in the prevalence of PCOS in different studies may be principally due to differences in the ethnic and geographic nature of the research populations (Daan *et al.*, 2014; Bil *et al.*, 2016).

IR results in a disturbance in glucose metabolism and often manifests as a prediabetes condition. There is an increasing amount of literature on the risk of developing gestational diabetes and type 2 diabetes due to IR in women with PCOS (Bayona *et al.*, 2022; Choudhury and Rajeswari, 2022; Livadas *et al.*, 2022). Gestational diabetes has been well defined in overweight and obese Western women but is less well documented in East Asian countries.

Although obesity is a typical feature of PCOS, lean individuals with PCOS are also at increased risk of IR-related issues. For example, Asian women with a lean PCOS phenotype have a higher prevalence of impaired glucose tolerance (IGT) than populations from America and Europe (Kakoly *et al.*, 2018). Thus, currently, available data highlight the importance of carefully evaluating IR and its consequences in the local clinical context, especially for individuals preparing for infertility treatment.

In lean individuals with PCOS, hyperinsulinemia is often evident postprandially but not in the fasted state (Morales *et al.*, 1996). Thus, fasting plasma glucose (FPG) often misses 40% of dysglycaemia and is inaccurate even in young, lean, normo-androgenaemic individuals (Ortiz-Flores *et al.*, 2019). Similarly, although reflecting average plasma glucose (PG) levels over a 2- to 3-month period, glycosylated haemoglobin (HbA1c) is a relatively poor diagnostic marker for diabetes, with a sensitivity of only 35% in women with PCOS (Velling Magnussen *et al.*, 2011). Therefore, the diagnosis of IGT in PCOS generally requires an oral glucose tolerance test (OGTT).

Type 2 diabetes and prediabetes are risk factors for increased rates of gestational diabetes, large for gestational age, congenital abnormalities, and pregnancy loss after infertility treatment (Wei *et al.*, 2017). Because type 2 diabetes and prediabetes are often asymptomatic, screening women with PCOS for type 2 diabetes and prediabetes has been recommended, but the best timing and method of screening remain unresolved (Rotterdam ESHRE/ASRM-Sponsored PCOS Consensus Workshop Group, 2004; Wild *et al.*, 2010; Legro *et al.*, 2013; Goodman *et al.*, 2015).

This study is designed to address this knowledge gap by investigating the impact of abnormalities of glucose metabolism on live birth outcomes in South-East Asian women with PCOS and infertility. Specifically, we assess whether glucose metabolism abnormalities influence live birth rates and pregnancy outcomes over a 24-month follow-up period. This will provide crucial insights for tailoring clinical management strategies in this unique patient population.

## Materials and methods

### Study design

This prospective cohort study was conducted at IVFMD, My Duc Hospital in Ho Chi Minh City, Vietnam between June 2020 and August 2024. The study was approved by the My Duc Hospital Ethics Review Board (approval number 08/20/ĐĐ-BVMD; date: 24 April 2020) and prospectively registered on clinicaltrials.gov (NCT04364087, 24 April 2020). All participants provided written informed consent.

### Study population

Eligible women were aged 18–40 years, had presented with infertility, and had been diagnosed with PCOS. PCOS was diagnosed when two out of the following three features were present: ovulation dysfunction (OD), clinical and/or biochemical signs of hyperandrogenism (HA), and polycystic ovarian morphology (PCOM) on ultrasound examination (Rotterdam ESHRE/ASRM-Sponsored PCOS Consensus Workshop Group, 2004). OD was determined based on an individual’s self-reported menstrual cycle (<21 or >35 days, or <8 cycles per year; >90 days for any one cycle; primary amenorrhoea by age 15 years or for >3 years postmenarche) (Teede *et al.*, 2018). Clinical HA was diagnosed based on the presence of at least one of the following: hirsutism (modified Ferriman–Gallwey [mFG] score of ≥3) or severe acne (Teede *et al.*, 2018). Biochemical HA was defined as a total testosterone level of ≥1.8 nmol/l or a free androgen index (FAI) of ≥6 (Teede *et al.*, 2018). Using transvaginal ultrasound transducers with a frequency bandwidth of 8 MHz, the threshold for PCOM was ≥20 follicles per ovary in either ovary and/or ovarian volume ≥10 ml, ensuring no corpora lutea, cysts, or dominant follicles were present (Teede *et al.*, 2018). Individuals with endocrine abnormalities, including thyroid-stimulating hormone (TSH) >5 mIU/ml, serum prolactin >30 μg/l, and any other concomitant endocrinopathy such as a history of hypothyroidism, Cushing’s syndrome, premature ovarian insufficiency, and late-onset or non-classic congenital adrenal hyperplasia were excluded.

### Comprehensive patient evaluation

#### First visit

All participants underwent routine evaluation of medical history, clinical examination, and transvaginal ultrasound. The diagnosis of PCOS was further classified into four clinical phenotypes, A (HA + OD + PCOM), B (HA + OD), C (HA + PCOM), or D (OD + PCOM), based on the National Institutes of Health 2012 recommendations (Evidence-based Methodology Workshop on Polycystic Ovary Syndrome (PCOS) | NICHD—Eunice Kennedy Shriver National Institute of Child Health and Human Development, 2012). Experienced physicians collected anthropometric data according to the standard study protocol, including body weight, height, waist and hip circumferences, waist-to-hip ratio (WHR), and BMI as per the World Health Organization (WHO) guidelines for Asian women. Trained midwives evaluated hirsutism and acanthosis nigricans, and fat mass was measured in the abdomen area using specific skinfold callipers (Accu-Measure^®^). The percentage of visceral adipose tissue for all patients was recorded using an electronic body composition scale (Tanita SC-330S). To minimize the influence of a meal, all anthropometric measurements were performed when the participant had been fasting for ≥4 h.

A fasting blood sample was obtained regardless of the timing in the menstrual cycle. LH (inter-assay coefficient of variation [CV] 2.3%), FSH (inter-assay CV 3.5%), oestradiol (inter-assay CV 2.7%), progesterone (inter-assay CV 6.2%), prolactin (inter-assay CV 5.2%), sex hormone-binding globulin (SHBG) (inter-assay CV 5.6%), and total testosterone (inter-assay CV 8.4%) were measured using the Elecsys technique, Cobas e411 system. FAI was calculated using the formula: FAI = [serum testosterone (in nmol/l)/serum SHBG (in nmol/l)] × 100. TSH (inter-assay CV 6.0%) and free thyroxine (fT4) (inter-assay CV 5.1%) were measured using the Access 2 immunoassay system, Beckman Coulter (Brea, CA, USA). High-density lipoprotein cholesterol (HDL-C) (inter-assay CV 2.4%), low-density lipoprotein cholesterol (LDL-C) (inter-assay CV 2.0%), and triglycerides (inter-assay CV 1.8%) were measured using the AU480 system (Beckman Coulter). Fasting serum insulin (inter-assay CV 2.8%) was measured using the Elecsys technique, Cobas e411 Analyzer. The Homeostasis Model Assessment of Insulin Resistance Index (HOMA-IR) was used to estimate IR. HOMA-IR was calculated as [FPG (in mmol/l) × fasting insulin (in μIU/ml)]/22.5 (Matthews *et al.*, 1985).

After a fast of ≥4 h, FPG (inter-assay CV 0.9%) was measured using the Beckman Coulter AU480 analyser, and HbA1c (inter-assay CV 1.00%) was measured using the Tosoh HLC-723GX analyser (Tokyo, Japan); participants who had not fasted for ≥4 h were asked to return for measurement of FPG the next day.

#### Second visit

Participants without known type 2 diabetes underwent an OGTT with 75 g of glucose. Individuals were recommended to maintain a regular diet for 3 days and then fast overnight for at least 8 h. Blood withdrawal (2 ml) was performed twice, once in the fasted state and again at 2 h after consumption of the glucose solution.

Participants were evaluated for type 2 diabetes and prediabetes based on the following criteria (American Diabetes Association, 2018):

- Type 2 diabetes: FPG ≥7.0 mmol/l and/or HbA1c ≥6.5% at any time point, or 2-h postprandial PG (2-h PG) ≥11.1 mmol/l during OGTT.
- Prediabetes: FPG 5.6–6.9 mmol/l, HbA1c 5.7–6.4%, and/or IGT. IGT was defined as 2-h PG between 7.8 and <11.1 mmol/l during OGTT.

The combined group diagnosed with type 2 diabetes or prediabetes was considered the abnormal glucose metabolism group, while the remaining group was the normal glucose metabolism group.

### Follow-up of pregnancy outcomes

After assessment of glucose metabolism, all patients who were identified as having a preconception glucose metabolism disorder (e.g. prediabetes or type 2 diabetes) were referred to an endocrinologist for specialized preconception care. This included lifestyle modifications and/or pharmacological interventions, aiming to optimize metabolic health before conception and potentially reduce the risk of adverse pregnancy outcomes. Conception was achieved through various methods, including natural conception, ovulation induction combined with IUI (OI/IUI), or IVF/IVM. These treatment options were counselled based on recommendations from the 2023 international evidence-based guideline (Teede *et al.*, 2023), in which first-line therapy involves optimizing preconception health and lifestyle, followed by OI with letrozole. Timed intercourse was recommended in the absence of male factor infertility, while IUI was offered in cases with mild male factors. IVF/IVM was indicated after failure of these initial approaches. Final decisions were individualized through shared decision-making, depending on clinical indications and patient preference (Teede *et al.*, 2023). Pregnancy outcomes, including rates of pregnancy and live birth, were tracked and documented over a 24-month follow-up period from the time of study enrolment.

### Outcomes

The primary outcome was the live birth rate at 24 months after enrolment into the study. Live birth was defined as the delivery of a live infant after 22 weeks of gestation, regardless of the method of conception (Zegers-Hochschild *et al.*, 2017). Gestational diabetes mellitus (GDM) was diagnosed according to the 2018 American Diabetes Association (ADA) one-step strategy (American Diabetes Association, 2019). A 75-g OGTT was performed at 24–28 weeks of gestation in women without type 2 diabetes diagnosed before the OGTT. Women with type 2 diabetes known before the first visit or diagnosed at the first or second preconception visit did not undergo OGTT. PG concentrations were measured at fasting and at 1 and 2 h after the glucose load, following an overnight fast of at least 8 h. GDM was diagnosed when any of the following PG levels were met or exceeded: fasting ≥92 mg/dl (5.1 mmol/l), 1-h ≥180 mg/dl (10.0 mmol/l), or 2-h ≥153 mg/dl (8.5 mmol/l) (American Diabetes Association, 2019). Hypertensive disorders of pregnancy (HDP), including gestational hypertension, preeclampsia, and eclampsia, were defined according to ACOG Practice Bulletin No. 222 (Gestational Hypertension and Preeclampsia, 2020). Neonatal complications, including congenital anomalies, were assessed by paediatric specialists at My Duc Hospital for infants delivered on-site. For infants delivered at other facilities, information was collected through structured phone interviews with the parents by the trained research staff.

### Statistical analyses

Data are presented using descriptive statistics: mean and SD for normally distributed variables, median and interquartile range for skewed variables, and number (%) for categorical variables. Univariable analysis was performed to identify factors associated with live birth at 24 months. Additional stratified analyses were performed to evaluate factors associated with live birth in patient subgroups based on the method of conception (natural, OI/IUI, or IVF/IVM). Separate univariate and multivariate logistic regression models were used to assess the association between glucose abnormalities and the risk of GDM and HDP. For analyses of GDM incidence, women with type 2 diabetes were excluded, whereas all participants were retained in the models assessing HDP. All variables with a *P*-value of ≤0.25 in univariate analysis were included in the subsequent multivariable analysis. All analyses were performed using the R statistical package (R version 3.6.1; R Foundation for Statistical Computing, Vienna, Austria), and a *P*-value of <0.05 was defined as statistically significant.

## Results

### Study population

**Figure 1.**
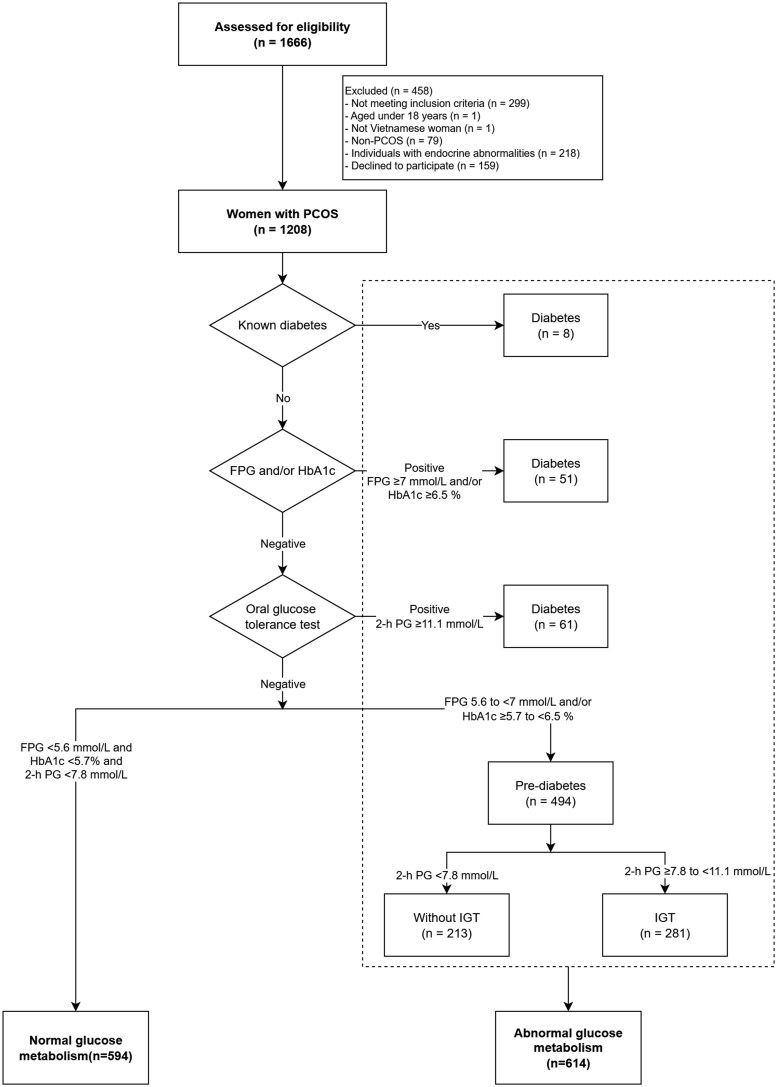
**Glucose metabolism testing procedure for women with polycystic ovary syndrome.** PCOS, polycystic ovary syndrome; FPG, fasting plasma glucose; HbA1c, glycosylated haemoglobin; IGT, impaired glucose tolerance; OGTT, oral glucose tolerance test; PG, plasma glucose.

A total of 1208 Vietnamese women with PCOS and infertility were enrolled in the study, including 594 (49.2%) with normal glucose metabolism and 614 (50.8%) with abnormal glucose metabolism of whom 120 (9.9%) had type 2 diabetes and 494 (40.9%) had prediabetes at baseline (Fig. 1, Table 1). Women in both groups were relatively young (mean age 28.8 ± 3.4 vs 29.7 ± 3.9 years, *P* < 0.001) and lean (mean BMI 21.8 ± 3.2 vs 24.2 ± 4.5 kg/m^2^, *P* < 0.001). Central obesity was more prevalent in the abnormal metabolism group, as reflected by higher waist circumference (74.9 ± 8.3 vs 79.9 ± 12.8 cm, *P* < 0.001), hip circumference (89.4 ± 6.6 vs 91.8 ± 11.3 cm, *P* < 0.001), and waist-to-hip ratio (0.8 ± 0.1 vs 0.9 ± 0.1, *P* < 0.001). Of the women who had preconception type 2 diabetes (n = 120), treatments included insulin (n = 18), metformin (n = 58), liraglutide (n = 22), and non-pharmacological therapy (n = 22). Of the 494 who had prediabetes before conception, treatments were metformin (n = 119), liraglutide (n = 11), and non-pharmacological therapy (n = 364). Insulin and metformin were continued during pregnancy as clinically indicated, whereas liraglutide was discontinued once pregnancy was confirmed.

**Table 1.** Participant demographic, anthropometric, and clinical characteristics at baseline.

Characteristics	Normal glucose metabolism (n = 594)	Abnormal glucose metabolism (n = 614)			***P*-value** *
		Type 2 diabetes (n = 120)	Prediabetes (n = 494)	Total (n = 614)	
Age, years	28.8 ± 3.4	30.5 ± 4.1	29.5 ± 3.9	29.7 ± 3.9	<0.001^†^
Age group, n (%)					<0.001^‡^
*<25 years*	52 (8.8)	7 (5.8)	44 (8.9)	51 (8.3)	
*25–30 years*	366 (61.6)	53 (44.2)	260 (52.6)	313 (51.0)	
*31–35 years*	157 (26.4)	48 (40.0)	153 (31.0)	201 (32.7)	
*>35 years*	19 (3.2)	12 (10.0)	37 (7.5)	49 (8.0)	
BMI, kg/m^2^	21.8 ± 3.2	26.3 ± 5.6	23.7 ± 4.0	24.2 ± 4.5	<0.001^†^
BMI category, n (%)					<0.001^‡^
*<18.5 kg/m^2^*	68 (11.4)	3 (2.5)	22 (4.5)	25 (4.1)	
*18.5–22.9 kg/m^2^*	366 (61.6)	29 (24.2)	228 (46.2)	257 (41.9)	
*23–24.9 kg/m^2^*	77 (13.0)	14 (11.7)	87 (17.6)	101 (16.4)	
*25–29.9 kg/m^2^*	68 (11.4)	49 (40.8)	120 (24.3)	169 (27.5)	
*≥ 30.0 kg/m^2^*	15 (2.5)	25 (20.8)	37 (7.5)	62 (10.1)	
Nulliparous, n (%)	411 (69.2)	86 (71.7)	340 (68.8)	426 (69.4)	0.94^‡^
Waist circumference, cm	74.9 ± 8.3	82.5 ± 20.8	79.3 ± 10.0	79.9 ± 12.8	<0.001^†^
Hip circumference, cm	89.4 ± 6.6	91.3 ± 21.0	91.9 ± 7.4	91.8 ± 11.3	<0.001^†^
Waist-to-hip ratio	0.8 ± 0.1	0.9 ± 0.2	0.9 ± 0.1	0.9 ± 0.1	<0.001^†^
Waist-to-hip category, n (%)					<0.001^‡^
*Central fat distribution (>0.85)*	236 (39.7)	92 (76.7)	283 (57.3)	375 (61.1)	
*Peripheral fat distribution (≤0.85)*	358 (60.3)	28 (23.3)	211 (42.7)	239 (38.9)	
Menstruation, n (%)					0.016^‡^
*Regular*	22 (3.7)	5 (4.2)	20 (4.0)	25 (4.1)	
*Irregular*	421 (70.9)	75 (62.5)	313 (63.4)	388 (63.2)	
*Amenorrhea*	151 (25.4)	40 (33.3)	161 (32.6)	201 (32.7)	
Hirsutism, n (%)	68 (11.4)	18 (15.0)	68 (13.8)	86 (14.0)	0.21^‡^
HA, n (%)	272 (45.8)	92 (76.7)	247 (50.0)	339 (55.2)	0.002^‡^
OD, n (%)	572 (96.3)	115 (95.8)	474 (96.0)	589 (95.9)	0.74^‡^
PCOM, n (%)	571 (96.1)	116 (96.7)	468 (94.7)	584 (95.1)	0.39^‡^
PCOS phenotype, n (%)					0.013^‡^
*A (HA + OD + PCOM)*	227 (38.2)	83 (69.2)	201 (40.7)	284 (46.3)	
*B (HA + OD)*	23 (3.9)	4 (3.3)	26 (5.3)	30 (4.9)	
*C (HA + PCOM)*	22 (3.7)	5 (4.2)	20 (4.0)	25 (4.1)	
*D (OD + PCOM)*	322 (54.2)	28 (23.3)	247 (50.0)	275 (44.8)	
Acanthosis nigricans n (%)	277 (46.6)	86 (71.7)	289 (58.5)	375 (61.1)	<0.001^‡^
Family history of diabetes, n (%)	113 (19.0)	42 (35.0)	129 (26.1)	171 (27.9)	<0.001^‡^
Family history of hypertension, n (%)	21 (3.5)	24 (20.0)	45 (9.1)	69 (11.2)	<0.001^‡^

Data are mean ± SD or number of patients (%).

* *P*-value for comparison between normal and abnormal glucose metabolism groups.

† *P*-values calculated using independent-samples *t-*tests.

‡ *P*-values calculated using chi-square tests.

HA, hyperandrogenism; OD, ovulatory dysfunction; PCOM, polycystic ovarian morphology.

Menstrual irregularities were common in both groups, with amenorrhea observed in 25.4% and 32.7% of participants in the normal and abnormal metabolism groups, respectively (*P* = 0.016). OD and PCOM were highly prevalent in both groups but rates of OD (96.3% vs 95.9%, *P* = 0.74) and PCOM (96.1% vs 95.1%, *P* = 0.39) were similar in the groups with normal versus abnormal glucose metabolism at baseline; the rate of HA was lower in the normal versus abnormal glucose metabolism group (45.8% vs 55.2%, *P* = 0.002). The most common PCOS phenotype was D in the normal glucose metabolism group (54.2%) and A in the abnormal glucose metabolism group (46.3%), while phenotypes B and C were uncommon in both groups, occurring in 3.9% and 3.7%, respectively, in the normal glucose metabolism group, and 4.9% and 4.1%, respectively, in the abnormal glucose metabolism group.

Acanthosis nigricans (46.6% vs 61.1%, *P* < 0.001), a positive history of diabetes (19.0% vs 27.9%, *P* < 0.001), and family history of hypertension (3.5% vs 11.2%, *P* < 0.001) were more common in the abnormal versus normal glucose metabolism group.

The endocrine and metabolic profile at baseline is summarized in Table 2. Compared to women with abnormal glucose metabolism, those with normal glucose metabolism had significantly higher LH levels, progesterone levels, LH/FSH ratio, and SHBG, but a lower FAI. Participants with normal glucose metabolism had a lower FPG, 2-h PG, fasting insulin, and HOMA-IR compared to those with abnormal glucose metabolism. Dyslipidaemia was more frequent in the group with abnormal versus normal glucose metabolism. Specifically, participants with normal glucose metabolism were less likely to have high triglycerides, high LDL-C, low HDL-C, and high total cholesterol compared to those with abnormal glucose metabolism.

**Table 2.** Biochemical profile of study participants at baseline.

Characteristics	Normal glucose metabolism (n = 594)	Abnormal glucose metabolism (n = 614)	*P*-value
AMH, ng/ml	9.3 ± 4.5	9.1 ± 4.4	0.5^†^
LH, U/l	13.8 ± 8.2	12.3 ± 6.9*	0.001^†^
FSH, U/l	6.2 ± 2.3	6.0 ± 2.0*	0.17^†^
Progesterone, nmol/l	1.2 ± 3.7	0.7 ± 2.4	0.003^†^
LH/FSH ratio	2.3 ± 1.1	2.1 ± 1.1*	0.005^†^
LH/FSH ratio group*, n (%)			0.011^‡^
*<1*	66 (11.1)	71 (11.7)	
*1–2*	179 (30.1)	229 (37.8)	
*>2*	349 (58.8)	306 (50.5)	
Total testosterone, nmol/l	1.5 ± 0.7	1.5 ± 0.7	0.25^†^
SHBG, mmol/l	48.6 ± 29.8	35.0 ± 25.8	<0.001^†^
FAI	4.2 ± 3.2	6.2 ± 4.8	<0.001^†^
Biochemical HA, n (%)	209 (35.2)	289 (47.1)	<0.001^‡^
TSH, U/l	1.9 ± 1.0	1.9 ± 1.0	0.48^†^
fT4, pmol/l	1.3 ± 1.4	1.2 ± 1.2	0.1^†^
Positive TPOAb, n (%)	70 (11.8)	71 (11.6)	0.99^‡^
Prolactin, μg/l	13.8 ± 6.2	13.0 ± 6.2	0.015^†^
FPG, mmol/l	5.0 ± 0.4	5.6 ± 1.3	<0.001^†^
Fasting insulin, mIU/l	17.0 ± 31.4	39.7 ± 79.7	<0.001^†^
HOMA-IR	3.8 ± 7.2	10.4 ± 23.6	<0.001^†^
HbA1c, %	5.3 ± 0.2	5.7 ± 0.7	<0.001^†^
2-h PG, mmol/l	6.1 ± 0.9	8.4 ± 2.1	<0.001^†^
Lipid profile, n (%)			
*Low HDL-C (<1 mmol/l)*	70 (11.8)	130 (21.2)	<0.001^‡^
*High LDL-C (>4.1 mmol/l)*	103 (17.3)	149 (24.3)	0.003^‡^
*High triglycerides (>2.3 mmol/l)*	89 (15.0)	219 (35.7)	<0.001^‡^
*High total cholesterol (>6.2 mmol/l)*	68 (11.4)	123 (20.0)	<0.001^‡^
Dyslipidaemia, n (%)	197 (33.2)	330 (53.7)	<0.001^‡^

* Data on LH and FSH were missing in 8 patients. Data are mean ± SD or number of patients (%).

† *P*-values calculated using independent-samples *t*-tests.

‡ *P*-values calculated using chi-square tests.

2-h PG, 2-h plasma glucose; AMH, anti-Müllerian hormone; FAI, free androgen index; FPG, fasting plasma glucose; fT4, free thyroxine; HA, hyperandrogenism; HbA1c, glycosylated haemoglobin; HDL-C, high-density lipoprotein cholesterol; HOMA-IR, homeostatic model assessment of insulin resistance; LDL-C, low-density lipoprotein cholesterol; SHBG, sex hormone-binding globulin; TPOAb, thyroid peroxidase antibody; TSH, thyroid-stimulating hormone.

### Outcomes

**Figure 2.**
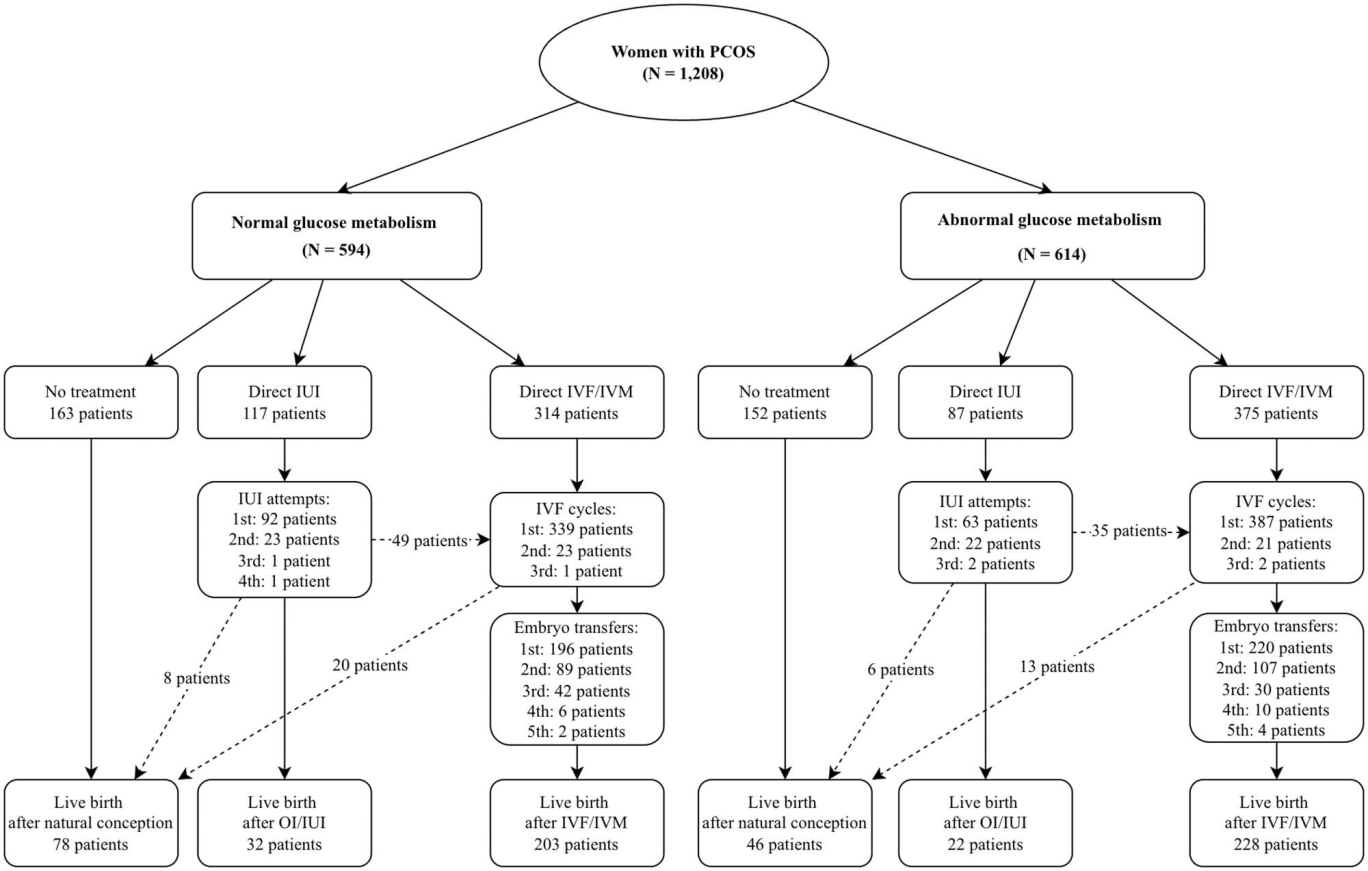
**Flowchart of treatment pathways and live birth outcomes over 24 months for women with polycystic ovary syndrome, stratified by baseline glucose metabolism.** PCOS, polycystic ovary syndrome; OI/IUI, ovulation induction combined with IUI.

The live birth rate at 24 months (primary outcome) was 50.4% (609/1208) in the overall study population (Fig. 2, Table 3). Live birth was achieved through natural conception in 34.3% of women (124/362), through OI/IUI in 50.9% (54/106), and through IVF/IVM in 58.2% (431/740). There was no significant difference in the live birth rate at 24 months between the normal and abnormal glucose metabolism groups (52.7% vs 48.2%, *P* = 0.12). Time to live birth was also comparable between these two groups.

**Table 3.** Reproductive outcomes at 24 months.

Characteristics	Normal glucose metabolism (n = 594)	Abnormal glucose metabolism (n = 614)	*P*-value
Live birth, n (%)	313 (52.7)	296 (48.2)	0.12^‡^
Time to live birth, days	370 [304; 477]	362 [301; 499]	0.8^§^
Induced abortion, n (%)	2 (0.3)	4 (0.7)	0.7^‡^
Gestational age at birth, weeks			
*Singleton*	38.1 ± 2.1	37.6 ± 2.9	0.04^†^
*Twins*	35.4 ± 3.1	34.8 ± 3.0	0.2^†^
Birth weight, grams			
*Singleton*	3163.8 ± 504.1	3118.4 ± 641.7	0.5^†^
*Twins*	1957.1 ± 566.2	2202.3 ± 668.4	0.3^†^
**Pregnancy complications, n (%)** *	**N = 338**	**N = 315**	
Miscarriages <12 weeks	54/594 (9.1)	59/614 (9.6)	0.83^‡^
Miscarriages 12 to <22 weeks	3 (0.9)	4 (1.3)	0.72^‡^
GDM	52 (15.4)	90 (28.6)	<0.001^‡^
HDP	8 (2.4)	29 (9.2)	<0.001^‡^
Antepartum haemorrhage	1 (0.3)	1 (0.3)	>0.9^‡^
Postpartum haemorrhage	1 (0.3)	0 (0.0)	>0.9^‡^
Preterm birth 22–28 weeks	4 (1.2)	5 (1.6)	0.74^‡^
Preterm birth 28–32 weeks	5 (1.5)	10 (3.2)	0.19^‡^
Preterm birth 32–37 weeks	36 (10.7)	45 (14.3)	0.19^‡^
**Neonatal complications, n (%)** **	**N = 342**	**N = 332**	
Neonatal ICU	40 (11.7)	48 (14.5)	0.3^‡^
Neonatal sepsis	6 (1.8)	9 (2.7)	0.4^‡^
Respiratory failure	22 (6.4)	24 (7.2)	0.7^‡^
Neonatal mortality	8 (2.3)	17 (5.1)	0.06^‡^
Congenital anomalies	4 (1.2)	5 (1.5)	>0.9^‡^

At the end of follow-up, 20 ongoing pregnancies in the normal glucose group and 11 in the abnormal glucose group had not yet reached a final pregnancy outcome. Data are mean ± SD, median [IQR], or number of patients (%).

* Number of women with ongoing pregnancy;

** Number of children.

† *P*-values calculated using independent-samples *t-*tests.

‡ *P*-values calculated using chi-squared tests.

§ *P*-values calculated using Mann–Whitney *U-*tests.

GDM, gestational diabetes mellitus; ICU, intensive care unit; HDP, hypertensive disorders of pregnancy.

Gestational complications, including GDM (28.0% vs 15.2%, *P* < 0.001) and HDP (9.0% vs 2.3%, *P* < 0.001) were more common in the abnormal versus normal glucose metabolism group. Rates of other pregnancy complications and neonatal complications did not differ significantly between the two groups. The overall incidence of congenital anomalies was low and was similar in women with normal and abnormal glucose metabolism. No specific pattern or clustering of anomalies was observed.

### Factors associated with live birth at 24 months

In the multivariate analysis, smaller waist circumference and larger hip circumference were associated with higher odds of live birth, whereas HA was associated with lower odds of live birth. Conception through OI/IUI or IVF/IVM was also associated with higher odds of live birth compared with natural conception (Table 4). When analyses were stratified by mode of conception (Supplementary Table S1), among women who conceived naturally, greater waist circumference and higher HOMA-IR were significantly associated with lower odds of live birth, whereas the presence of HA was associated with higher odds of live birth. After conception through IVF/IVM, higher BMI was the only factor that remained significantly associated with lower odds of live birth. There were no factors significantly associated with live birth after conception through OI/IUI.

**Table 4.** Factors associated with live birth at 24 months.

Characteristics	No live birth (N = 599)	Live birth (N = 609)	OR (95% CI)	
			Univariate	Multivariate
Age, years	29.2 ± 4.0	29.3 ± 3.4	1.01 (0.98; 1.04)	–
BMI, kg/m^2^	23.6 ± 4.5	22.4 ± 3.6	0.93 (0.91; 0.96)	0.96 (0.91; 1.01)
Nulliparous, n (%)	415 (69.3)	422 (69.3)	1.00 (0.78; 1.28)	–
Waist circumference, cm	79.0 ± 12.2	75.9 ± 9.7	0.97 (0.96; 0.98)	0.97 (0.94; 0.99)
Hip circumference, cm	91.4 ± 10.4	89.9 ± 8.2	0.98 (0.97; 1.00)	1.04 (1.01; 1.06)
HA, n (%)	340 (56.8)	271 (44.5)	0.61 (0.49; 0.77)	0.74 (0.58; 0.96)
OD, n (%)	570 (95.2)	591 (97.0)	1.66 (0.92; 3.09)	1.42 (0.76; 2.73)
PCOM, n (%)	571 (95.3)	584 (95.9)	1.14 (0.66; 2.00)	–
Family history of diabetes, n (%)	146 (24.4)	138 (22.7)	0.91 (0.70; 1.19)	–
Family history of hypertension, n (%)	55 (9.2)	35 (5.7)	0.60 (0.39; 0.93)	0.86 (0.53; 1.40)
AMH, ng/ml	9.1 ± 4.4	9.2 ± 4.6	1.01 (0.98; 1.03)	–
LH/FSH ratio	2.3 ± 1.1	2.2 ± 1.1	0.91 (0.82; 1.01)	0.92 (0.83; 1.03)
HOMA-IR	8.0 ± 19.9	6.3 ± 15.3	0.99 (0.99; 1.00)	1.00 (0.99; 1.01)
HbA1c, %	5.5 ± 0.7	5.4 ± 0.6	0.91 (0.76; 1.08)	–
2-h PG, mmol/l	6.8 ± 2.6	6.9 ± 2.3	1.02 (0.97; 1.06)	–
Low HDL-C (<1 mmol/l), n (%)	105 (17.5)	96 (15.8)	0.88 (0.65; 1.19)	–
High LDL-C (>4.1 mmol/l), n (%)	132 (22.0)	120 (19.7)	0.87 (0.66; 1.15)	–
High triglycerides (>2.3 mmol/l), n (%)	172 (28.7)	137 (22.5)	0.72 (0.56; 0.93)	0.89 (0.67; 1.18)
High total cholesterol (>6.2 mmol/l), n (%)	97 (16.2)	94 (15.4)	0.94 (0.69; 1.29)	–
Method of conception, n (%)*				
*Natural*	238 (39.7)	124 (20.4)	Ref.	Ref.
*OI/IUI*	52 (8.7)	54 (8.9)	1.99 (1.28; 3.09)	2.68 (2.05; 3.52)
*IVF/IVM*	309 (51.6)	431 (70.8)	2.67 (2.06; 3.48)	1.80 (1.15; 2.82)

* Method of conception was defined as the final intervention during the follow-up period. Data are mean ± SD or number of patients (%). 2-h PG, 2-h plasma glucose; AMH, anti-Müllerian hormone; HA, hyperandrogenism; HbA1c, glycosylated haemoglobin; HDL-C, high-density lipoprotein cholesterol; HOMA-IR, homeostatic model assessment of insulin resistance; LDL-C, low-density lipoprotein cholesterol; OD, ovulatory dysfunction; OI/IUI, ovulation induction combined with IUI; OR, odds ratio; PCOM, polycystic ovarian morphology.

### Factors associated with GDM and HDP

In univariate and multivariable logistic regression models that included maternal age, BMI, family history of diabetes, family history of hypertension, and preconception glucose metabolism (Table 5), increased maternal age, higher BMI, and abnormal preconception glucose metabolism were associated with higher odds of developing GDM. For HDP, only increased maternal age and abnormal preconception glucose metabolism were significantly associated with higher odds.

**Table 5.** Factors associated with gestational diabetes mellitus and hypertensive disorders of pregnancy.

Characteristics	**GDM (N = 599)** *		**HDP (N = 653)** **	
	Univariate OR (95% CI)	Multivariate OR (95% CI)	Univariate OR (95% CI)	Multivariate OR (95% CI)
Age, years	1.11 (1.05; 1.17)	1.10 (1.04; 1.16)	1.14 (1.04; 1.25)	1.12 (1.03; 1.23)
BMI, kg/m^2^	1.11 (1.05; 1.17)	1.08 (1.02; 1.14)	1.15 (1.06; 1.25)	1.09 (0.99; 1.19)
Family history of diabetes (Yes)	1.18 (0.75; 1.84)	–	1.92 (0.92; 3.82)	1.41 (0.66; 2.89)
Family history of hypertension (Yes)	0.86 (0.31; 2.03)	–	4.16 (1.56; 9.78)	2.27 (0.78; 6.03)
Glucose metabolism				
*Normal*	Ref.	Ref.	Ref.	Ref.
*Abnormal*	2.84 (1.93; 4.22)	2.48 (1.66; 3.73)	4.12 (1.93; 9.87)	3.02 (1.37; 7.35)

* Number of ongoing pregnant women without type-2 diabetes;

** Number of ongoing pregnant women.

GDM, gestational diabetes mellitus; OR, odds ratio; HDP, hypertensive disorders of pregnancy.

## Discussion

We observed a high prevalence of abnormal glucose metabolism in infertile Vietnamese women with PCOS, 40.9% of whom had prediabetes and 9.9% had type 2 diabetes. This finding is notable due to the fact that most women in our cohort were lean, indicating that metabolic dysfunction in PCOS is not limited to individuals with obesity. These findings highlight a metabolic vulnerability that is often overlooked in lean Asian women with PCOS. Numerically, the live birth rate over the 24 months after the first visit was slightly lower in women with versus without abnormal glucose metabolism (52.7% vs 48.2%) but the between-group difference was not statistically significant (*P* = 0.12). Abnormal glucose metabolism was independently associated with an increased risk of developing GDM and HDP.

The prevalence of abnormal glucose metabolism observed in our study was higher than rates reported in previous Asian studies. For example, a large Chinese cohort found that 17.9% of women with PCOS had prediabetes and 3.6% had diabetes (Wen *et al.*, 2024), while rates of 30.9% and 7.1%, respectively, were reported in a South Asian cohort (Ullah *et al.*, 2022). These variations in the prevalence of abnormal glucose metabolism likely reflect the impact of ethnic and population factors, and highlight the importance of tailored screening for Asian women with PCOS. It is possible that the comprehensive detection strategies used in our study contributed to the higher number of abnormal glucose metabolism cases detected. This included the combined use of FPG, HbA1c, and OGTT, which provides greater diagnostic sensitivity than single-test strategies, as supported by the 2023 International Evidence-based PCOS Guideline (Teede *et al.*, 2023).

In terms of reproductive outcomes, our study adds to the limited body of current literature by prospectively evaluating how preconception glucose abnormalities influence pregnancy outcomes in women with PCOS. Our findings are consistent with data from a secondary analysis of a multicentre randomized controlled trial including 1508 women with PCOS, which showed that women with preconception IGT had higher risks of GDM, large-for-gestational-age, and pregnancy loss, independent of BMI (Wei *et al.*, 2017). Taken together, these findings support a role for abnormal preconception glucose metabolism as a risk factor for obstetric complications such as GDM and HDP. Other studies have also explored the relationship between IR and reproductive outcomes, showing that elevated markers such as the triglyceride-glucose-body mass index (TyG-BMI), triglyceride-glucose index (TyG), and HOMA-IR are negatively associated with live birth rates in IVF cycles. This supports the hypothesis that dysglycaemia has an adverse effect on reproductive potential (Li *et al.*, 2024; Wu *et al.*, 2025).

The link between abnormal glucose metabolism and adverse reproductive and obstetric outcomes in women with PCOS is likely multifactorial. IR plays a central role, leading to compensatory hyperinsulinemia that stimulates ovarian androgen production and disrupts follicular maturation and ovulation (Zhang *et al.*, 2019). IR also induces oxidative stress and chronic inflammation, impairing oocyte competence and embryo development (Katsiki *et al.*, 2009; Jiang *et al.*, 2023). Endothelial dysfunction secondary to hyperinsulinemia and hyperglycaemia may contribute to vasoconstriction and hypertension through reduced nitric oxide availability and increased sympathetic activity (Kayemba-Kay’s *et al.*, 2013; Lin *et al.*, 2021). In addition, abnormal glucose metabolism can impair placental development and insulin signalling, resulting in placental hypoxia and maladaptation, which can further predispose to individuals to GDM and hypertensive disorders (Tano *et al.*, 2023; Gallo and Savoia, 2024).

In our multivariate analyses, smaller waist circumference and larger hip circumference were significantly and positively associated with achieving live birth, whereas HA was significantly and negatively associated with live birth. In stratified analyses by mode of conception, greater waist circumference and higher HOMA-IR index were associated with lower odds of live birth, whereas HA was associated with higher odds among women who conceived naturally. In women conceiving through IVF/IVM, increased BMI was the only factor negatively associated with live birth, and no significant associations were observed after OI/IUI. Our findings reinforce the role of central adiposity, IR, and BMI as key contributors to impaired reproductive outcomes. They also suggest that metabolic dysfunction, rather than obesity alone, plays a central role in the pathogenesis of pregnancy complications and subfertility in women with PCOS.

The results of the current study have important clinical implications for the management of women with PCOS in reproductive settings. The high prevalence of abnormal glucose metabolism, even among lean women, underscores the need for routine and comprehensive metabolic screening before conception using a combination of FPG, HbA1c, and OGTT. This integrated approach, in line with the 2023 International PCOS Guideline (Teede *et al.*, 2023), improves diagnostic sensitivity and helps to detect mild glucose abnormalities that individual tests may miss. Early identification of metabolic abnormalities would allow timely interventions such as lifestyle modification, weight management, and (if indicated) pharmacological therapy, which have been shown to improve metabolic health and pregnancy outcomes (Liu *et al.*, 2021; Abdalla *et al.*, 2022; Teede *et al.*, 2023; Cheshire *et al.*, 2025).

Even with comprehensive preconception care, women with glucose abnormalities in our study remained more likely to develop GDM and HDP, indicating that current strategies may need further refinement for high-risk women. This highlights the need for enhanced, individualized strategies, potentially combining dietary counselling, structured exercise programs, and insulin-sensitizing agents, to optimize outcomes. In addition, central adiposity, IR and BMI, which were identified in subgroup analyses as factors associated with a lower live birth rate, should be recognized as accessible and clinically relevant markers for metabolic risk assessment. Integrating these parameters into preconception evaluation may help stratify risk, guide counselling, and tailor management plans to improve both fertility and pregnancy outcomes.

One strength of our study was the large sample size. However, there was no formal sample size calculation for the primary endpoint and statistical power may therefore not have been adequate to detect significant between-group differences in the live birth rate. In addition, because most of the women in our study conceived after IVF/IVM, the natural and OI/IUI conception subgroups were small which may also have limited statistical power for the subgroup analyses. Therefore, findings from these subgroups should be interpreted with caution. Another strength is that the 24-month follow-up period enabled comprehensive tracking of both spontaneous and assisted conceptions, offering detailed insights into fertility trajectories. Additionally, the inclusion of anthropometric indices and OGTT allowed for a comprehensive assessment of glucose metabolism and its relationship with reproductive outcomes. However, several other limitations should be acknowledged. First, the current findings are derived from a South-East Asian population with a relatively lean BMI profile, and therefore require validation in other ethnic groups and clinical settings before broader generalization. Second, although our total cohort included 1208 women, the number of pregnancies available for evaluating live birth and obstetric outcomes was smaller, again limiting statistical power. Third, as noted above, the proportion of women conceiving via the different methods was not balanced, which may have introduced selection bias and resulted in differences in patient characteristics between these subgroups. Finally, metabolic assessments were only performed at baseline, preventing evaluation of longitudinal changes and their dynamic effects on reproductive outcomes. Future studies should consider multicentre designs and repeated metabolic assessments to better capture temporal changes and their influence on outcomes.

In conclusion, while abnormal glucose metabolism did not significantly affect the live birth rate at 24 months, it was associated with a significantly higher risk of developing GDM and HDP. These findings support routine preconception metabolic screening and tailored fertility management in women with PCOS. Early detection and individualized preconception management may help reduce pregnancy complications and improve overall reproductive outcomes. Interventions targeting obesity, IR, and other metabolic dysfunctions may remain central components of fertility care in women with PCOS, given their potential contribution to metabolic risk and pregnancy complications.

## Supplementary Material

hoag012_Supplementary_Data

## Data Availability

The data underlying this article will be shared on reasonable request to the corresponding author.
