# Supplementary material for: Impact of glucose metabolism abnormalities on live birth rate in South-East Asian women with polycystic ovary syndrome
Source: Hum Reprod Open. 2026 Feb 17;2026(2):hoag012. doi: 10.1093/hropen/hoag012 (PMC12981221; doi:10.1093/hropen/hoag012)
Supplement: hoag012_Supplementary_Data [file hoag012_supplementary_data.docx]

**Supplementary Table S1. Factors associated with live birth at 24 months, subgroup by method of conception**

| Characteristics | Natural  (N = 362) | | OI/IUI  (N = 106) | | IVF/IVM  (N = 740) | |
| --- | --- | --- | --- | --- | --- | --- |
|  | **Univariate**  **OR**  **(95%CI)** | **Multivariate**  **OR**  **(95%CI)** | **Univariate**  **OR**  **(95%CI)** | **Multivariate**  **OR**  **(95%CI)** | **Univariate**  **OR**  **(95%CI)** | **Multivariate**  **OR**  **(95%CI)** |
| Age, years | 1.03  (0.97; 1.09) | - | 1.01  (0.90; 1.15) | - | 0.97  (0.93; 1.01) | 0.97  (0.93; 1.01) |
| BMI, kg/m^2^ | 0.92  (0.87; 0.97) | 1.04  (0.91; 1.20) | 0.95  (0.86; 1.06) | - | 0.93  (0.89; 0.97) | 0.94  (0.88; 0.99) |
| Nulliparous (yes) | 0.71  (0.44; 1.15) | 0.74  (0.45; 1.23) | 0.67  (0.25; 1.73) | - | 1.29  (0.95; 1.76) | 1.33  (0.96; 1.85) |
| Waist circumference, cm | 0.96  (0.94; 0.98) | 0.93  (0.87; 0.98) | 0.99  (0.94; 1.03) | - | 0.98  (0.96; 0.99) | 0.98  (0.95; 1.01) |
| Hip circumference, cm | 0.97  (0.94; 0.99) | 1.05  (0.98; 1.12) | 1.01  (0.95; 1.06) | - | 0.99  (0.97; 1.00) | 1.03  (1.00; 1.06) |
| HA (Yes) | 5.76  (1.11; 142) | 5.91  (1.04; 111.67) | 1.98  (0.16; 63.6) | - | 1.32  (0.63; 2.74) | - |
| OD (Yes) | 0.80  (0.30; 2.27) | - | - | - | 1.17  (0.57; 2.37) | - |
| PCOM (Yes) | 0.47  (0.30; 0.73) | 0.68  (0.41; 1.10) | 0.86  (0.40; 1.85) | - | 0.64  (0.47; 0.85) | 0.73  (0.53; 1.00) |
| Family history of diabetes (Yes) | 0.99  (0.60; 1.62) | - | 0.74  (0.25; 2.06) | - | 0.94  (0.66; 1.33) | - |
| Family history of hypertension (Yes) | 0.40  (0.11; 1.09) | 1.15  (0.29; 3.84) | 0.48  (0.06; 2.74) | - | 0.63  (0.37; 1.06) | 0.92  (0.52; 1.63) |
| AMH, ng/mL | 0.98  (0.93; 1.03) | - | 1.05  (0.96; 1.14) | - | 1.01  (0.98; 1.04) | - |
| LH/FSH ratio | 1.02  (0.83; 1.25) | - | 1.01  (0.71; 1.44) | - | 0.91  (0.80; 1.03) | 0.88  (0.77; 1.01) |
| HOMA-IR | 0.93  (0.88; 0.98) | 0.96  (0.91; 0.99) | 0.97  (0.93; 1.01) | - | 1.00  (0.99; 1.01) | - |
| HbA1c, % | 0.61  (0.36; 1.04) | 1.31  (0.68; 2.41) | 0.46  (0.13; 1.60) | - | 0.97  (0.80; 1.19) | - |
| 2-h PG, mmol/L | 0.98  (0.89; 1.08) | - | 0.95  (0.79; 1.14) | - | 1.03  (0.97; 1.09) | - |
| Low HDL-C (<1 mmol/L) | 0.61  (0.31; 1.12) | 1.06  (0.50; 2.19) | 0.96  (0.27; 3.36) | - | 0.98  (0.67; 1.44) | - |
| High LDL-C (>4.1 mmol/L) | 0.85  (0.49; 1.47) | - | 1.08  (0.39; 3.02) | - | 0.82  (0.58; 1.17) | - |
| High triglycerides (>2.3 mmol/L) | 0.38  (0.21; 0.67) | 0.52  (0.26; 1.01) | 0.69  (0.21; 2.19) | - | 0.85  (0.62; 1.18) | - |
| High total cholesterol (>6.2 mmol/L) | 0.86  (0.44; 1.60) | - | 1.28  (0.43; 3.93) | - | 0.88  (0.60; 1.31) | - |

*2-h PG: 2-hour plasma glucose; AMH: anti-Müllerian hormone; aOR: adjusted odds ratio; CI: confidence interval; HA: hyperandrogenism; HbA1c: glycosylated haemoglobin; HDL-C: high-density lipoprotein cholesterol; HOMA-IR: Homeostatic Model Assessment of Insulin Resistance; IVF/IVM: in-vitro fertilisation/in-vitro maturation; LDL-C: Low-density lipoprotein cholesterol; OD: ovulatory dysfunction; OI/IUI: ovulation induction combined with intrauterine insemination; PCOM: polycystic ovarian morphology.*
